# Assessing Retention in Care and Factors Associated With Antiretroviral Therapy Among People Living With HIV in the Upper West Region of Ghana: A Retrospective Cohort Study

**DOI:** 10.1155/arat/8819023

**Published:** 2025-12-17

**Authors:** Henry Ping-Naah, Stephen Ayisi Addo, Ekow Wiah, Marijanatu Abdulai, Kwadwo Owusu, Damien Punguyire, Shamwill Issah, Bismark Sarfo

**Affiliations:** ^1^ Department of Epidemiology and Disease Control, School of Public Health, University of Ghana, P.O. Box LG 13, Legon, Accra, Ghana, ug.edu.gh; ^2^ Municipal Hospital, Ghana Health Service, P.O. Box 19, Lawra, Upper West Region, Ghana, ghanahealthservice.org; ^3^ National AIDS/STI Control Programme, P.O.Box Kb 547 Korle-Bu, Accra, Ghana; ^4^ Regional Health Administration, Ghana Health Service, P.O. Box 298, Wa, Upper West Region, Ghana, ghanahealthservice.org; ^5^ The Palladium Group, Tackling Deadly Diseases in Africa Program (TDDAP2), 35 Patrice Lumumba Street Airport, Accra, Ghana

**Keywords:** ART, Ghana, people living with HIV, retention, Upper West Region

## Abstract

**Background:**

Antiretroviral therapy (ART) programs that retain people living with HIV (PLWH) in care are essential for maintaining viral suppression, improving health outcomes, and halting the spread of HIV. While ART accessibility has improved, retention in care is still low in Ghana. Although several factors account for this, there are limited data to support it. This study assessed the factors influencing retention in care of PLWH in the Upper West Region (UWR) of Ghana.

**Method:**

The study used a retrospective cohort design to track 482 PLWH who started ART in 2019 across 14 ART clinics in the UWR of Ghana. Demographic, clinical, and psychological elements linked to retention were accessed by extracting data from the HIV electronic tracker database. The characteristics of the study participants summarized using descriptive statistics, estimates of retention rates, and relationships between presumed predictors and retention were obtained through Cox proportional hazards regression and Kaplan–Meier survival analysis.

**Results:**

The results demonstrate that 384/482 (79.7%) participants remained in care at 6 months, 354/482(73.4%) at 12 months, 298/482 (61.8%) at 24 months, and 260/482 (53.9%) at 36 months, indicating a decline in ART retention with time. Age, HIV status disclosure, and viral load suppression are important determinants of retention. Older ages between 50 and 79 years (HR = 0.29, 95% CI: 0.13–0.61) have a 71% reduced risk of attrition compared with younger age groups. Psychosocial factors were positively associated with retention as the HIV status disclosure had HR = 0.50 (95% CI: 0.29–0.88). The unknown viral load status significantly increased the risk of attrition (HR = 6.41, 95% CI: 4.50–9.12).

**Conclusion:**

This study has demonstrated that retention rates decrease with time and that retention was significantly predicted by age and viral load status, with better retention rates shown in older PLWH and those aware of their viral load status.

## 1. Introduction

In order to achieve the Sustainable Development Goal (SDG) objective 3.3, which demands of ending the AIDS epidemic, there is a need to improve antiretroviral therapy (ART) accessibility, which is key to improving people living with HIV (PLWH) prognoses and lowering transmission risks and deaths [1]. One major factor that could mitigate against achieving SDG 3.3 is retention in the care of PLWH. According to UNAIDS [2], it is estimated that 350,000 (310,000–420,000) people in Ghana are living with an HIV prevalence of 1.7% (1.4%–2.0%).

ART increases survival by reducing virus replication to levels below detection. To significantly reduce new HIV infections globally, UNAIDS has set ambitious treatment targets known as Project 95‐95‐95, aiming to achieve 95% of PLWH knowing their status, 95% of diagnosed individuals on ART, and 95% of those on ART achieving viral suppression by 2030 [2, 3].

ART retention is a major problem in low‐ and middle‐income nations. Since patient adherence to ART is crucial, there are worries regarding PLWH retention [4]. The ART retention rate among HIV patients in Ghana was 70% over 3 years [5].

In the Upper West Region (UWR) of Ghana, the ART program faces some challenges, which contribute to inadequate 36‐month retention rates of 58% among PLWH [6].

In other regions such as the Ashanti Region of Ghana, Kweku et al. [5] found that 70.3% of PLWH were retained in ART for up to 3 years. Other studies in other parts of Africa predicted a retention rate of 35.7% among adolescents living with HIV (ALWH) in the remote South Western Ugandan district of Ibanda [7].

Several factors are attributed to retention in ART programs. These factors include demographic factors, socioeconomic challenges, health system‐related diseases, and psychosocial factors. Factors associated with greater odds of retention in care in the Ashanti Region of Ghana are being employed, female sex, divorced status, and primary level of education [5]. Meanwhile, the factors that could influence retention in care of PLWH in the UWR of Ghana are not known. Bershteyn et al. [8] analyzed data from South Africa, Malawi, and sub‐Saharan Africa and emphasized the importance to improve the strategies of ART retention, especially in populations at greatest risk. The consequences of suboptimal retention in the ART program may compromise PLWH health outcomes, increase the risk of transmission, potentially develop drug resistance, and stress public health resources.

This study assessed retention in care and factors associated with ART among PLWH in treatment in the UWR of Ghana. The outcome of this study holds the potential to inform targeted interventions and strategies aimed at improving retention rates and eventually enhancing HIV treatment programs and the overall well‐being of PLWH.

## 2. Methods

### 2.1. Study Area

**Figure 1:**
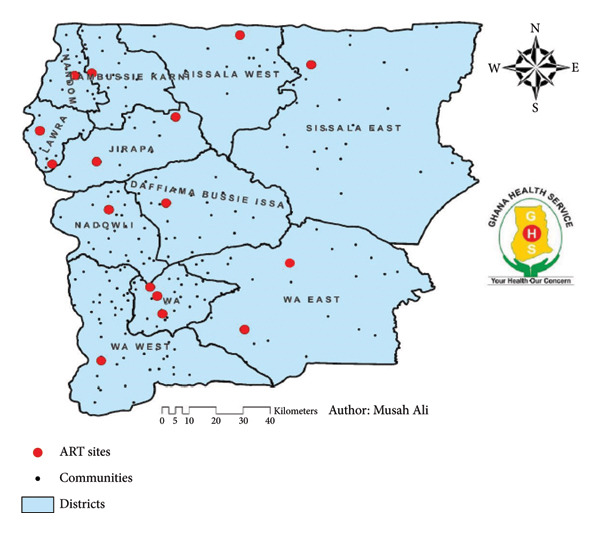
UWR map of ART sites.

This study was conducted in 14 ART clinics in the UWR (Figure 1), which is situated in the northwestern part of Ghana. It borders with Northern Region of Ghana to the south, Burkina Faso to the north and west, and the UWR of Ghana to the east. The area is defined by geographical coordinate’s longitudes 1°25′ W and 2° 45″ and latitudes 9° 30″ N and 11° N and features Black Volta and White Volta rivers (Figure 1). From Ghana’s 2021 Population and Housing Census (PHC), the region’s projected population is 901,502.

The district health systems are managed by District Health Management Teams (DHMTs), supervised by the Regional Health Management Team (RHMT). There are over 559 facilities that provide HIV services. Out of the 559 facilities, 10 are hospitals, 5 polyclinics, 18 clinics, 75 health centers, and 450 CHPS compound. The region has 16 facilities that offer ART services, 18 facilities providing Differentiated Service Delivery (DSD), and 7 viral load (VL) testing sites. The region recorded an HIV prevalence of 2.5% in the 2021 HIV Sentinel Survey [9]. PLWH on ART are provided with ART in 16 ART facilities with a maximum of three permanent staff per ART clinic. There is high rural and urban migration among the youth, which has an impact on ART retention due to the tropical climate with one rainy season.

The main economic activity is peasant farming. It is a mix of urban and rural settings with houses built in scattered settlements. The main transportation in the region is motorcycles, tricycles, bicycles, and occasionally donkeys.

### 2.2. Operational Definition

Retention in ART care: In this study, retention in ART care was defined as PLWH who remained alive and were receiving ART and had at least one documented clinic visit or drug refill within 90 days.

Lost to follow‐up (LTFU): PLWH who missed their scheduled ART appointment for more than 90 consecutive days without documented death were classified as being LTFU.

### 2.3. Enrollment of Participants

#### 2.3.1. Inclusion Criteria

PLWH who initiated ART in 2019 at the 14 ART facilities and received clinical care follow‐up in the UWR were included.

#### 2.3.2. Exclusion Criteria

The exclusion criteria are as follows:

1. PLWH who were initiated on ART in different regions and transferred to the UWR to continue ART were excluded from the study.
2. PLWH who stopped ART at some point and restarted were excluded to avoid bias in estimating the retention duration, which would require longitudinal modeling and re‐entry analysis.

### 2.4. Sample Size Estimation

**Figure 2:**
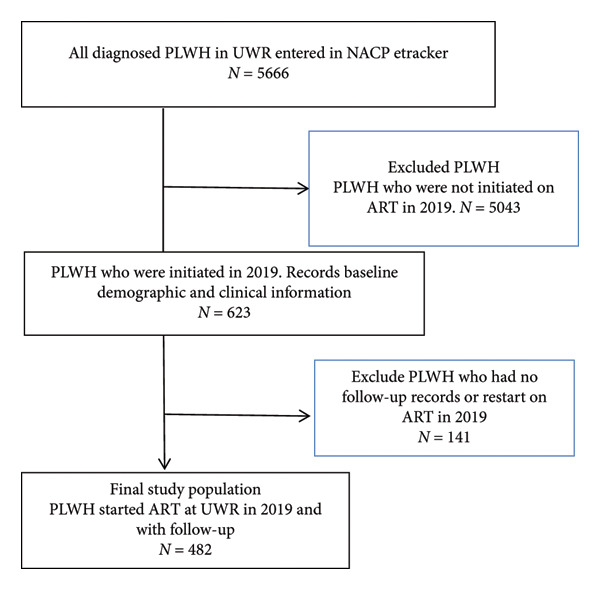
Schematic diagram of the selection of the study subjects.

The census sample technique was used in this study to include all eligible members of the target population. In the UWR, 5666 PLWH were first extracted from the National AIDS/STD Control Programme (NACP) e‐tracker database. The study did, however, restrict its scope to those who started ART in 2019. PLWH were reduced by 5043 through an exclusion process, leaving 623 PLWH who began ART in 2019. Further criteria were used to ensure the data quality. Specifically, 141 PLWH who had no follow‐up records or restarted ART in 2019 were not included in the study, leaving 482 PLWH in total (Figure 2). A census technique was used to obtain retention factors for this cohort. Figure 2 shows the selection process of PLWH.

### 2.5. Data Management and Analysis

Data were extracted from the NACP e‐tracker into Microsoft Excel version 2010 for data organization. The data were further analyzed for completeness, duplicates, and consistency. Stata (version 15.0) was used to analyze the data. Descriptive statistics were used to provide summaries of demographic characteristics, including age, sex, education level, and marital status. Retention rates at 6, 12, 24, and 36 months on ART were calculated. Retention was defined as PLWH who continued engagement with ART for at least six months and was calculated as the proportion of PLWH retained in care and expressed as a percentage for months 6, 12, 24, and 36. Measures such as mean, median, standard deviation, and percentiles were used to describe the distribution of variables. Kaplan–Meier survival analysis was employed to estimate retention probabilities over a 36‐month follow‐up period. Kaplan–Meier curves were generated for the entire study population and for specific subgroups based on demographic, clinical, and psychosocial variables.

Multivariable Cox proportional hazards regression analysis was conducted to identify factors associated with retention in ART. Independent variables, including demographic characteristics (age, sex, and marital status), clinical (VL suppression and comorbidity), and psychosocial (HIV status disclosed) variables, were included in the model. Hazard ratios (HRs), which show the relative risk of attrition from ART associated with each independent variable while adjusting for other factors, were estimated using the Cox proportional hazards model [10].

### 2.6. Ethical Approval

Ethical approval was obtained from the Ghana Health Service Ethics Review Committee with Approval Number GHS‐ERC: 051/04/24. Permissions were granted by the UWR Health Service and facilities heads to use their facilities data for the study. Participants’ records were de‐identified by removing names and client program unique identification numbers in the dataset before analysis, and the confidentiality of participants’ records was maintained.

## 3. Results

### 3.1. Descriptive of Demographic Characteristics and Clinical Variables

A total of 482 PLWH were involved in the study. The study excluded 141 newly diagnosed PLWH who had no follow‐up records or restarted ART in 2019 during the sample size estimation. With the initial sample size of 623, the 141 represent 22.6% of the initial sample size, which is an indication of high attrition. The mean age of PLWH at registration was 33.6 years with a standard deviation of 2.6 years. The age distribution was 1 year at the lowest and 79 years at the highest with an interquartile range (IQR) of 26–41. Males made up 25.3% of PLWH (122/482), with females making up the majority (74.7%, 360/482). The majority of PLWH (55.0%, 265/482) were married, with the next largest group being single (18.0%, 86/482). Nearly half of the participants had no formal education (49.2%, 237/482). The occupations of PLWH were variedly distributed with the largest being farming or fishing (37.0%, 178/482). Other common occupations included traders or shop assistants (0.6%, 51/482) and business owners (10.0%, 48/482) (Table 1).

**Table 1:** Demographic characteristics of PLWH.

Characteristics	Number (%)
Age at initiation, years	
1–14	26 (5.0)
15–24	70 (15.0)
25–49	340 (70.5)
50–79	46 (9.5)
Sex	
Male	122 (25.3)
Female	360 (74.7)
Marital status	
Married	265 (55.0)
Single	86 (18.0)
Cohabiting	9 (1.9)
Widowed	48 (10.0)
Divorced	15 (3.1)
Separated	12 (2.5)
No response	23 (4.8)
Not applicable	24 (5.0)
Formal education level	
None	237 (49.2)
Preschool	7 (1.5)
Primary	73 (15.2)
Middle school level certificate	5 (1.0)
Junior high school	62 (12.9)
Senior high school/vocational/technical	53 (11.0)
Tertiary	23 (4.8)
No response	22 (4.6)
Occupation	
Business	48 (10.0)
Hairdresser	2 (0.4)
Farming/fishing	178 (37.0)
Housewife	27 (5.6)
Professional	32 (6.6)
Seamstress	6 (1.2)
Pupil/student	51 (10.6)
Trader/shop assistant	20 (4.2)
Others	118 (24.5)

The majority of PLWH (94.8%, 457/482) pick the ARVs at the nearest ART clinic to their residents. A little above half (54.4%, 262/482) of PLWH have disclosed their HIV status to a partner, relative, or adherence monitor. Out of the 482 PLWH, 47.3% (228/482) had data for VL testing, of which 89.9% had suppressed VL. Comorbidity was as low as 0.62% (3/482) among PLWH in this study.

### 3.2. Analysis of Retention Rate on ART at 6, 12, 24, and 36 Months

**Figure 3:**
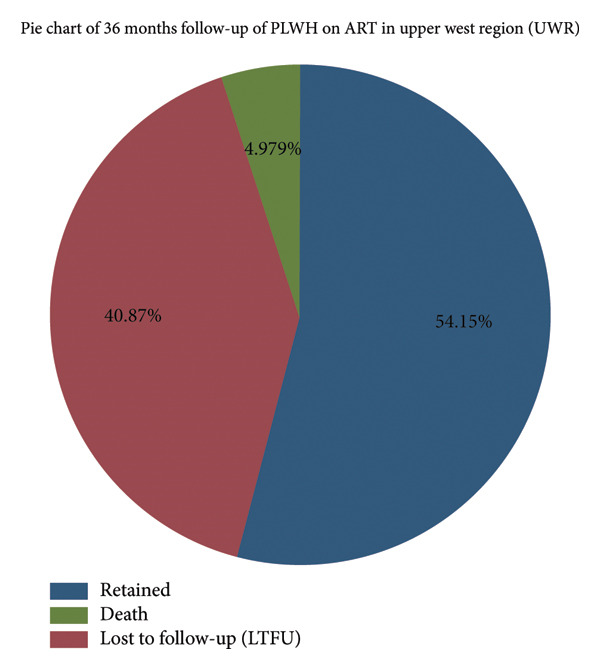
Pie chart of 36‐month follow‐up of PLWH on ART in UWR.

A total of 482 PLWH were included in the analysis of retention rates at 6‐, 12‐, 24‐, and 36‐ months after ART initiation. On average, PLWH remained in care for about 2 years (mean = 24.7 months, Std. dev. = 14.1). The retention rate at 6 months in care was 79.7% (384/482), while 20.3% (98/482%) were either LTFU or had died. This means nearly 80% for the first 6‐month retention rate in ART programs. Retention at 12 months was 73.4% (354/482) with 26.6% (128/482) LTFU or dead. This is a decline in retention of nearly 6% about the 6‐month follow‐up. By 24 months, the retention rate had further decreased to 61.8% (298/482) with 38.2% (184/482%) LTFU or dead. The increased number of LTFU or dead affected more than one‐third of the cohort. During the 36‐month follow‐up, there was a further decline in the retention rate to 54.15% (261/482) with 40.87% (197/482) being LTFU and 4.98% (24/482) dead (Figure 3).

### 3.3. Kaplan–Meier Survival Curves Analysis for Retention Rates

**Figure 4:**
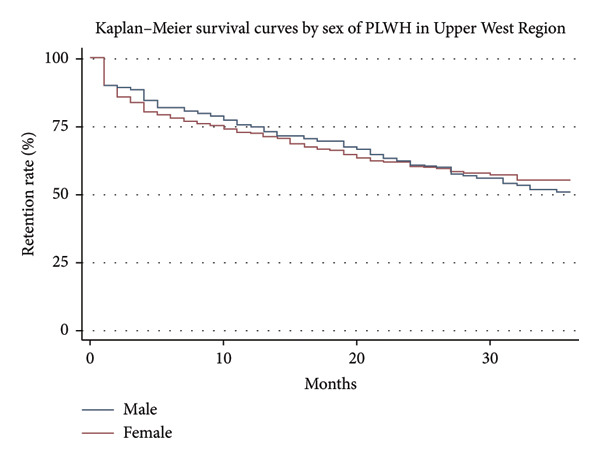
Kaplan–Meier survival curve by sex of PLWH in UWR.

Figure 4 shows the retention rates for males and females of PLWH in the UWR over 36 months. Females exhibited a marginally greater retention rate than males during the observation period, according to the survival curves. There were not many differences between the attrition rates on the curves for the two sexes, especially in the early part of the follow‐up (Figure 4). Male and female retention rates converge with time, culminating in comparable attrition percentages at the 36‐month mark. By the end of the study period, almost 55.0% of females and 50.8% of males were still in care. Figure 4 shows the curves by sex.

### 3.4. Kaplan–Meier Survival Curve by Age Group

**Figure 5:**
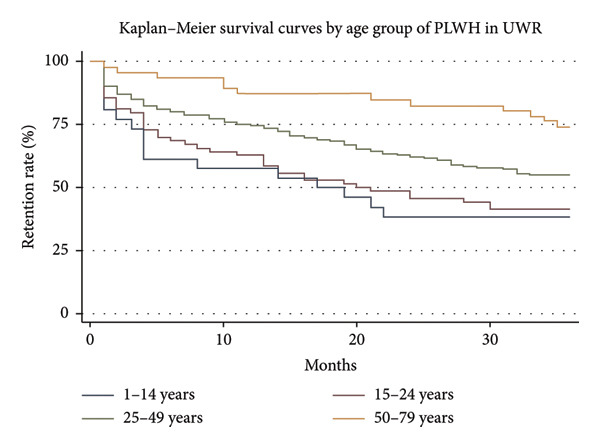
Kaplan–Meier survival curve by age group of PLWH in UWR.

Retention rates among PLWH for 36 months vary significantly according to age, as shown in the Kaplan–Meier survival curves (Figure 5) and table data. The age group 50–79 years has the highest retention rate (73.91%), and it declines gradually, showing a steady retention in care. There was a relatively high early drop‐off and a modest retention rate of 55% for the 25–49 age group. Younger age groups, particularly those between 1 and 14 and 15–24 years, have the lowest retention rates (41.43% and 38.46%, respectively), with sharp early decreases as shown in Figure 5.

### 3.5. Kaplan–Meier Survival Curves by V L Group in UWR

**Figure 6:**
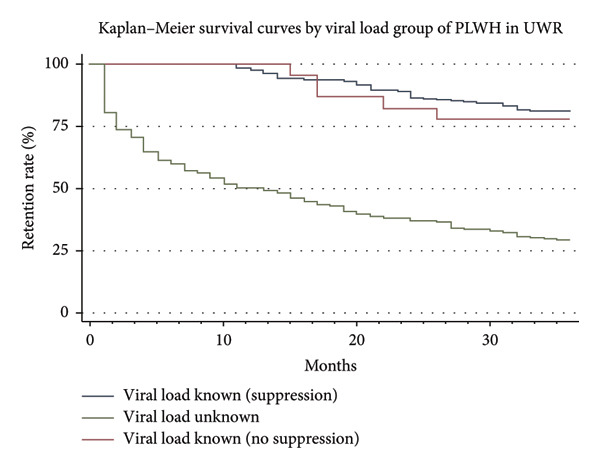
Kaplan–Meier survival curve by viral load group in UWR.

The Kaplan–Meier survival curves (Figure 6) show the status of the VL for the 36 months among PLWH. The highest retention rate (81.5%) was seen in PLWH with known VL suppression, closely followed by people with known VL but no suppression (78.3%). By comparison, the group whose VL was unknown showed a sharp drop in retention; at the end of 36 months, only 29.5% of them were retained (Figure 6).

### 3.6. Cox Proportional Hazards Regression Analysis to Assess Factors Associated With Retention

#### 3.6.1. Analysis to Assess Demographic Factors Associated With Retention

The age of PLWH was grouped into four categories: 1–14, 15–24, 25–49, and 50–79 years (Table 2). The age group 1–14 years was used as a reference group. The 15–24 age group had no statistical significance with an HR of 0.89 (95% CI: 0.50–1.58; *p* = 0.685). In those between 25 and 49 years of age, there was an HR of 0.59 (95% CI: 0.35–0.99; *p* = 0.044), showing a reduced risk of attrition by 41%. At age 50–79 years, HR was 0.29 (95% CI: 0.134–0.61; *p* ≤ 0.001), showing 71% lower hazard of attrition from care. Sex did not significantly affect retention in care, with males having an HR of 0.93 (95% CI: 0.70–1.26; *p* = 0.653). Retention in ART did not depend on the sex of the participants. The marital status contributed little to the variations in retention and was not significant: Single HR: 1.18 (95% CI: 0.83–1.67; *p* = 0.363), cohabiting HR: 0.63 (95% CI: 0.20–1.99; *p* = 0.433), widowed HR: 0.60 (95% CI: 0.35–1.03; *p* = 0.062), and divorced HR: 0.37 (95% CI: 0.12–1.15; *p* = 0.085). Education level showed no association with retention. Preschool and primary education were not associated with retention with HRs of 1.59 (95% CI: 0.58–4.31; *p* = 0.367) and 1.13 (95% CI: 0.77–1.67; *p* = 0.535), respectively. The middle and high levels of schooling showed no significance as well. Occupation was also not significant. Farming or fishing had an HR of 0.61 (95% CI: 0.32–1.15; *p* = 0.124), and housewives had an HR of 0.51 (95% CI: 0.22–1.20; *p* = 0.125), neither of which was statistically significant (Table 2).

**Table 2:** Univariate and multivariate analysis of Cox proportional hazards regression result**s**.

Variables	Univariate			Multivariate		
	HR	95% CI	*p* value	HR	95% CI	*p* value
Age group						
1–14	1.0			1.0		
15–24	0.89	(0.498, 1.581)	0.685	0.20	(0.042, 0.996)	0.049
25–49	0.59	(0.352, 0.985)	0.044	0.14	(0.028, 0.745)	0.021
50–79	0.29	(0.137, 0.612)	0.001	0.08	(0.013, 0.449)	0.004
Sex						
Female	1.0			1.0		
Male	0.93	(0.695, 1.256)	0.653	1.08	(0.783, 1.499)	0.629
Marstatus (marital status)						
Married	1.0			1.0		
Single	1.18	(0.830, 1.665)	0.363	0.81	(0.526, 1.256)	0.350
Cohabiting	0.63	(0.201, 1.987)	0.433	0.44	(0.134, 1.432)	0.172
Widowed	0.60	(0.351, 1.026)	0.062	0.70	(0.396, 1.221)	0.206
Divorced	0.37	(0.116, 1.150)	0.085	0.36	(0.113, 1.153)	0.085
Separated	1.19	(0.555, 2.546)	0.657	0.99	(0.440, 2.213)	0.974
Missing data	1.84	(1.089, 3.094)	0.022	1.02	(0.413, 2.505)	0.972
Formedulevl (education level)						
None	1.0			1.0		
Preschool	1.59	(0.583, 4.308)	0.367	1.04	(0.335, 3.259)	0.940
Primary	1.13	(0.766, 1.670)	0.535	0.93	(0.614, 1.408)	0.730
Middle school (MSLC)	0.44	(0.061, 3.142)	0.412	0.54	(0.073, 3.966)	0.542
Junior high	1.37	(0.925, 2.035)	0.115	1.26	(0.814, 1.950)	0.300
Senior high	1.14	(0.735, 1.768)	0.559	0.95	(0.568, 1.577)	0.834
Tertiary	0.97	(0.506, 1.860)	0.928	0.65	(0.308, 1.368)	0.256
Missing data	2.24	(1.321, 3.804)	0.003	2.75	(0.973, 9.500)	0.109
Occupation						
Others	1.0			1.0		
Business	0.50	(0.234, 1.052)	0.068	0.52	(0.234, 1.173)	0.116
Hairdresser	2.90*e* − 15	(0.0)	1.000	7.23*e* − 17	(0.0)	1.000
Farming/fishing	0.61	(0.324, 1.146)	0.124	0.65	(0.317, 1.330)	0.237
Housewife	0.51	(0.217, 1.204)	0.125	0.48	(0.190, 1.205)	0.118
Professional	0.79	(0.371, 1.691)	0.547	0.92	(0.394, 2.136)	0.843
Seamstress	0.19	(0 0.025, 1.506)	0.117	0.20	(0.025, 1.610)	0.131
Pupil/student	1.48	(0.680, 3.228)	0.322	1.29	(0.490, 3.400)	0.086
Trader/shop assistant	0.50	(0.236, 1.058)	0.070	0.49	(0.218, 1.105)	0.086
Unemployed	0.75	(0.370, 1.540)	0.439	0.80	(0.369, 1.755)	0.585
Not applicable	1.03	(0.461, 2.295)	0.946	1.96	(0.195, 19.624)	0.568
Missing data	1.00	(0.474, 2.125)	0.993	0.42	(0.128, 1.412)	0.163

In multivariate analysis, age remained an important factor in retention; the age group 15–24 years had an HR of 0.20 (95% CI: 0.04–1.00; *p* = 0.049), meaning that they were 80% less likely to suffer from attrition than their older PLWH. The subgroup analysis by age indicated that those aged 25–49 had an HR of 0.14 (95% CI: 0.03–0.75; *p* = 0.021) i.e., an 86% lesser likelihood for attrition compared to those aged 50–79 who had an HR of 0.08 (95% CI: 0.01–0.45; *p* = 0.004), meaning that they were 92% less likely to experience attrition. Sex was also not a significant factor of retention in the multivariate model. Males had an HR of 1.08 (95% CI: 0.783–1.50; *p* = 0.629), showing not much difference in retention with females after controlling for other factors. Being single shows no statistically significant association with attrition, with an HR of 0.81 (95% CI: 0.53–1.26; *p* = 0.350), while cohabiting had an HR of 0.44 (95% CI: 0.13–1.43; *p* = 0.172), indicating a lower risk of attrition, though not statistically significant. Widowed individuals showed a lower risk of attrition (HR = 0.70; 95% CI: 0.40–1.22; *p* = 0.206), while divorced PLWH had an even lower risk (HR = 0.36; 95% CI: 0.11–1.15; *p* = 0.085), though neither was statistically significant. Additionally, individuals with missing marital status data had a higher risk of attrition (HR = 1.02; 95% CI: 0.41–2.51; *p* = 0.972). Education level did not show significant associations with retention in the multivariate model. PLWH with tertiary education had an HR of 0.65 (95% CI: 0.31–1.37; *p* = 0.256), though not statistically significant. Missing data for education were associated with a higher risk of attrition (HR = 2.75; 95% CI: 0.97–9.50; *p* = 0.109), suggesting that incomplete information may indicate a greater risk of dropping out of care. The analyses for occupation remained nonsignificant in the multivariate analysis. PLWH in business had an HR of 0.52 (95% CI: 0.23–1.17; *p* = 0.116), and those in farming or fishing had an HR of 0.65 (95% CI: 0.32–1.33; *p* = 0.237), both of which were not statistically significant. Housewives had an HR: 0.48 (95% CI: 0.19–1.21; *p* = 0.118), which was also not significant. However, students had an HR of 1.29 (95% CI: 0.49–3.40; *p* = 0.607), indicating a higher risk of attrition, though not statistically significant (Table 2).

#### 3.6.2. Assess Clinical Factors Associated With Retention in Care Among PLWH

On the effect of clinical factors such as VL monitoring (suppression status) and comorbidities, univariate analysis was carried out to assess their impact on retention in care. VL status is a significant factor in whether PLWH remains in care or not. Specifically, PLWH with an unknown VL had a very high HR of 6.41 (95% CI: 4.50–9.12; *p* < 0.001), showing that they were more than six times likely to result in attrition compared to those whose VL was known and suppressed. For those with known VLs but not suppressed, their HR: 1.20 (95% CI: 0.47–3.04; *p* = 0.705) was not statistically significant (Table 3). It would suggest that even without viral suppression, PLWH who know their viral status do not have a significantly higher hazard of attrition compared to PLWH with suppressed VLs. Comorbidities were not associated with retention in care. PLWH who did not have comorbidities had an HR of 0.81 (95% CI: 0.20–3.27; *p* = 0.771), meaning that the attrition risk is not much different than for those with comorbidities.

**Table 3:** Results of clinical factors of the Cox proportional hazard model analysis.

Variables	Univariate			Multivariate		
	HR	95% CI	*p* value	HR	95% CI	*p* value
Comorb (comorbidity)						
Yes	1.0 (ref)			1.0 (ref)		
No	0.81	(0.202, 3.273)	0.771	1.13	(0 0.281, 4.562)	0.861
vlgroup (viral load group)						
Viral load known(suppression)	1.0 (ref)			1.0 (ref)		
Viral load known (no suppression)	1.20	(0.471, 3.042)	0.705	1.20	(0.471, 3.040)	0.706
Viral load unknown	6.41	(4.503, 9.116)	0.001	6.41	(4.506, 9.124)	0.001

VL status in predicting outcomes was confirmed through multivariate analysis. The HR among those with an unknown VL remained at 6.41 (95% CI: 4.51–9.12; *p* < 0.001), which shows that PLWH who did not know their VL was significantly more likely to drop out of care, even after controlling for the comorbidity factor. This only confirms what the univariate analysis indicated that VL is central to retention. For PLWH who knew their VL but were not suppressed, the HR in multivariate analysis was 1.20 (95% CI: 0.47–3.04; *p* = 0.706); there was no increase in the attrition risk. In other words, they are most likely to keep attending if they are informed, even though their VL is not suppressed. The multivariate analysis shows again that the presence of comorbidity was not a significant factor of attrition; as in the univariate analysis, no significant difference was seen in that regard. The HR of comorbidity against having no comorbidity is 1.13 (95% CI: 0.28–4.56, *p* = 0.861), showing that it is not a factor for attrition (Table 3).

#### 3.6.3. Assess the Association Between Psychosocial Factors With Retention in Care

The univariate analysis shows that the HIV status disclosure is a significant factor in retention in care (Table 4). The HR was 0.50 (95% CI: 0.28–0.88; *p* = 0.016) for those who had disclosed their HIV status, meaning that they were 50% less likely to experience attrition than those who had not. PLWH who did not disclose had a very small reduction in risk of retention with an HR of 0.95 (95% CI: 0.54–1.65; *p* = 0.843) and was not significant (Table 4). This finding implies that disclosing one’s HIV status is essential for enhancing care retention. The analysis also evaluated access to ART at the nearest ART clinic, albeit this factor did not reveal significant results. In comparison to PLWH who did not pick ART at the nearest ART clinic, those who had access to ART at the closest clinic had an HR of 1.14 (95% CI: 0.62–2.09; *p* = 0.674), indicating that there was no difference in the chance of attrition between those who pick treatment at the nearest ART clinic and who did not.

**Table 4:** Results of psychosocial factors of the Cox proportional hazard model analysis.

Variables	Univariate			Multivariate		
	HR	95% CI	*p* value	HR	95% CI	*p* value
Accessart (access to ART at the nearest ART clinic)						
No	1.0 (ref)			1.0 (ref)		
Yes	1.14	(0.621, 2.088)	0.674	1.15	(0.626, 2.106)	0.654
Hivstatdisc (HIV status disclosure)						
Not applicable	1.0 (ref)			1.0 (ref)		
Disclosed	0.50	(0.286, 0.878)	0.016	0.50	(0.286, 0.880)	0.016
Not disclosed	0.95	(0.542, 1.648)	0.843	0.95	0.543, 1.652)	0.848

HIV status disclosure continued to be a significant factor of retention in care in the multivariate analysis (Table 4). The HR for PLWH who disclosed the HIV status was 0.50 (95% CI: 0.29–0.88; *p* = 0.016), and for those who did not disclosed the HIV status, the HR was 0.95 (95% CI: 0.54–1.65; *p* = 0.848) with a small, reduced risk of retention. Disclosed HIV status was statistically significant and is in line with the univariate findings and emphasizes how important the idea that the disclosure of HIV status had a protective effect on retention. The multivariate analysis’s findings regarding access to ART at the closest ART clinic matched those of the univariate model. The HR of 1.15 (95% CI: 0.63–2.11; *p* = 0.654) for patients who had access to ART at the nearest clinic was not statistically significant (Table 4). This result suggests that retention in care was not independently factored by geographic proximity to ART services.

## 4. Discussion

The exclusion of 141 newly diagnosed PLWH without follow‐up records or restarted ART in 2019 shows a critical early attrition point consistent with challenges reported under the test‐and‐treat approach, where immediate ART initiation sometimes results in poor early retention, which impacts viral suppression. Participants LTFU may discontinue their medication and be unable to monitor their VLs, which could hinder progress toward achieving viral suppression in 95% of PLWH. This poses a significant threat to the realization of the UNAIDS 95‐95‐95 targets.

This study has demonstrated that retention rates declined with time, with 79.7% of PLWH retained in care after 6 months and 53.9% at 36 months. According to previous studies, PLWH’ retention in care decreases over time, from about 80% at 12 months to about 45%–54% at 36 months [11, 12]. The reduction in retention rate underscores the necessity of focused interventions aimed at addressing PLWH dropout‐causing variables, including transportation challenges, stigma, and the accessibility of psychosocial support services [13].

Analysis of the data from this study revealed that the likelihood that PLWH will stay in care is significantly influenced by their age and status about VL suppression. Those between the ages of 15 and 24 had an 80% lower chance of attrition than those in older age groups, while those between the ages of 25 and 49 had an even lower risk. Mohammed et al. [14] and Bielick et al. [15] in their study also found older age to be associated with better retention and viral suppression. This pattern implies that elderly PLWH are more likely to remain involved in their care, maybe because of the improved life stability and healthcare literacy. Younger PLWH, particularly those under the age of 25, revealed lower retention rates, which may reflect limited caregiver support structures and the psychosocial challenges of children and adolescence. Previous studies [16, 17] showed that strong caregiver or family involvement enhances adherence and reduces attrition among younger populations.

Another crucial clinical condition was the state of one’s VL; those who had no idea what their VL was had a much greater attrition rate. A study by Mokhele et al. [18] also found that VL awareness is crucial, as those unaware of their status have higher attrition rates. However, comparing VLs suppressed and unsuppressed PLWH did not show differences retention. The findings show that VL monitoring, rather than viral suppression, is a determinant of retention. PLWH with unknown VL results were significantly more likely to disengage from care, implying that routine monitoring and feedback mechanisms, rather than suppression alone, promote sustained retention. The univariate and multivariate studies did not find any statistical significance in the retention prediction of other characteristics, including sex, occupation, and educational attainment.

These results are in line with several studies in sub‐Saharan African, which have highlighted the value of VL disclosure and monitoring in enhancing ART retention. The idea that retention in care is closely linked to awareness of health status is supported, for example, by Muhula et al. [19], who reported that interventions to monitor VL and provide consistent feedback to PLWH significantly enhanced retention rates and could contribute to achieving the UNAIDS 95‐95‐95 targets by 2030. Furthermore, Cloete et al. [20] showed that proper clinical management, result accessibility, and VL testing rates were all markedly enhanced by a comprehensive intervention centered on VL management in South Africa. Younger age groups, especially adolescents and young adults, also have lower viral suppression outcomes with high tendency of stopping ART than other age groups [16, 17]. This finding was also evident in the current study, which found that younger participants had a lower retention rate during the 36‐month duration of care.

This study did not find any statistically significant association between marital status, education, and occupation; however, age and VL did emerge as crucial determinants in retention. Neither marital status nor education level showed statistically significant relationships with retention in the multivariate analysis. This finding runs counter to some previous research [21, 22] that revealed a high correlation between these characteristics and care retention. This disparity might result from the UWR’s rural location, where socioeconomic variables such as education and employment may have less of an immediate impact on health‐seeking behavior than in more urbanized areas.

PLWH who declared their HIV status had a much higher likelihood of staying in care, according to the data. Their HR showed that they were 50% less likely to experience attrition than those who had not disclosed their status. This result is consistent with the research, which shows that disclosure is frequently linked to improved adherence to ART regimens, less stigma, and higher social support [23, 24]. Nonetheless, PLWH who decide not to reveal their status could rely on alternative supports or coping strategies, like continuing with their private treatment regimen. This implies that while encouraging disclosure is crucial, it must be carried out in a way that upholds the privacy and autonomy of PLWH.

Meanwhile, retention rates were unaffected by the availability of ART at the closest clinic. The study shows no difference in the likelihood of participants staying in care between those who could access close ART facilities and those who had to travel further for treatment. The study by Monroe et al. [25], which indicated that retention rates were not significantly impacted by access to neighboring ART providers, supports this conclusion. The lack of significance in this study may suggest that, even in cases where ART services are geographically accessible, other factors such as stigma, the cost and mode of transportation, and financial constraints may have a greater impact on whether PLWH stay in care.

The importance of psychosocial support for retention, particularly about HIV status disclosure, needs more research. The qualitative components of disclosure, such as the contribution of family and community support to keeping PLWH in care, should direct future research. Further investigation on the use of VL testing services is necessary, as evidenced by the significant attrition rate among individuals whose viral levels are unknown. Investigating if making these services more accessible could lower attrition and boost program retention would be helpful.

The results of this investigation highlight how crucial age and suppressed VLs are to keeping PLWH in treatment. A clear knowledge of their VL status and older age are major predictors of continued engagement in care, which improves health outcomes and lowers the risk of transmission. These findings support the necessity for customized treatments that highlight the difficulties younger PLWH and those with less access to VL testing facilities experience. All things considered, improving care retention will necessitate a multimodal strategy that tackles clinical and psychosocial retention hurdles in ART programs.

Even though the study’s conclusions are unique to the UWR of Ghana, they offer insightful information that can be applied to other rural and resource‐constrained environments. Therefore, the results are still quite relevant for similar programmatic circumstances, even though broader generalization should be done with caution.

## 5. Conclusions

The study shows that retention rates decrease with time, reaching 53.9% at 36 months, which is similar to research conducted in sub‐Saharan Africa. Retention was significantly predicted by age and VL status, with better retention rates shown in older PLWH and those aware of their VL. Clinical characteristics such as comorbidities and psychosocial factors such as access to ART at the nearest clinic did not significantly predict retention. These results highlight how important it is to implement tailored strategies that target younger PLWH and those who are unsure of their VL status to improve retention.

## Ethics Statement

Ethical approval was obtained from the Ghana Health Service Ethics Review Committee with Approval Number GHS‐ERC: 051/04/24. Permissions were granted by the UWR Health Service and facilities heads to use their facilities data for the study. Participants’ records were de‐identified by removing names and client program unique identification numbers in the dataset before analysis, and confidentiality of participants’ records was maintained.

## Consent

Consent and permission were obtained for the publication of these data from the participants and the health facilities.

## Disclosure

All authors read and approved the final manuscript.

## Conflicts of Interest

The authors declare no conflicts of interest.

## Author Contributions

Henry Ping‐Naah made contributions to the study conception, design, acquisition of data, and analysis including interpretation of the data and drafting of the manuscript. Stephen Ayisi Addo, Ekow Wiah, Marijanatu Abdulai, Kwadwo Owusu, Damien Punguyire, and Shamwill Issah contributed to data interpretation and validation and review and editing of the manuscript. Bismark Sarfo contributed to the study conception and design, drafting of the manuscript, and interpretation of the data and critically reviewed the manuscript for intellectual content.

## Funding

No funding was received for this research.

## Data Availability

The data that support the findings of this study are available on request from the corresponding author. The data are not publicly available due to privacy or ethical restrictions.

## References

[1] Lopez M. , Overcoming Barriers to Antiretroviral Access: Global Perspectives, Journal of Infectious Diseases. (2024) 16, no. 2.

[2] UNAIDS , Global HIV Statistics People Living With HIV People Living With HIV Accessing Antiretroviral Therapy New HIV Infections AIDS-Related Deaths, 2024, Fact Sheet.

[3] Boakye D. S. and Adjorlolo S. , Achieving the UNAIDS 95-95-95 Treatment Target by 2025 in Ghana: A Myth or a Reality?, Global Health Action. (2023) 16, no. 1, 10.1080/16549716.2023.2271708. PMC10627043 37921654

[4] Nosyk B. and Humphrey L. , Highlighting the Need for Investment and Innovation in ART Retention Interventions, Lancet Global Health. (2022) 10, no. 9, e1218–e1219, 10.1016/s2214-109x(22)00327-8. 35961333 PMC10370491

[5] Kweku R. O. , Anto B. P. , Atakorah J. , Frimpong E. , and Morgan R. , Retention in Care, Loss to Follow-Up and Associated Patient Characteristics: A Retrospective Cohort Study Among Adults Receiving Antiretroviral Therapy From Urban Health Facilities in Ghana, Clinical Journal of HIV & AIDS. (2021) 5, no. 1, 70–75, 10.36959/695/574.

[6] NACP Assessment DSD Performance Review Report, 2023, https://NACPRepository.

[7] Gray K. L. , Kiazolu M. , Jones J. et al., Liberia Adherence and Loss-to-Follow-Up in HIV and AIDS Care and Treatment: A Retrospective Cohort of Adolescents and Adults From 2016–2019, PLOS Global Public Health. (2022) 2, no. 3, 10.1371/journal.pgph.0000198. PMC10021315 36962289

[8] Bershteyn A. , Jamieson L. , Kim H.-Y. et al., Transmission Reduction, Health Benefits, and Upper-Bound Costs of Interventions to Improve Retention on Antiretroviral Therapy: A Combined Analysis of Three Mathematical Models, Lancet Global Health. (2022) 10, no. 9, e1298–e1308, 10.1016/s2214-109x(22)00310-2. 35961353 PMC9380252

[9] Ghana Health Service , Annual Report–National AIDS/STI Control Programme (NACP), 2025, Ghana Health Service, https://nacpghana.com/bitstreams/4b6437c8-0295-4238-891e-426fe49249de/download.

[10] Ezeilo C. I. , Umeh E. U. , Osuagwu D. C. , and Onyekwere C. K. , Exploring the Impact of Factors Affecting the Lifespan of HIVs/AIDS Patient’s Survival: An Investigation Using Advanced Statistical Techniques, Open Journal of Statistics. (2023) 13, no. 04, 595–619, 10.4236/ojs.2023.134029.

[11] Mee P. , Rice B. D. , Lemsalu L. et al., Changes in Patterns of Retention in HIV Care and Antiretroviral Treatment in Tanzania Between 2008 and 2016: An Analysis of Routinely Collected National Programme Data, Journal of Global Health. (2019) 9, no. 1, 10.7189/jogh.09.010424, 2-s2.0-85064968738. PMC6445500 30992984

[12] Carlucci J. G. , Liu Y. , Clouse K. , and Vermund S. H. , Attrition of HIV-Positive Children From HIV Services in Low- and Middle-Income Countries: A Systematic Review and Meta-Analysis, AIDS. (2019) 33, no. 14, 2375–2386, 10.1097/QAD.0000000000002366, 2-s2.0-85072048049. 31764102 PMC6905128

[13] Moges N. A. , Olubukola A. , Micheal O. , and Berhane Y. , HIV Patients Retention and Attrition in Care and Their Determinants in Ethiopia: A Systematic Review and Meta-Analysis, BMC Infectious Diseases. (2020) 20, no. 439, 10.1186/s12879-020-05168-3. PMC7310275 32571232

[14] Mohammed D. Y. , Koumoulos L. M. , Martin E. G. , and Slim J. , Annual and Durable HIV Retention in Care and Viral Suppression Among Patients of Peter Ho Clinic, 2013-2017, PLoS One. (2020) 15, no. 12, 10.1371/journal.pone.0244376. PMC7771864 33373385

[15] Bielick C. , Canan C. E. , Ingersoll K. , Waldman A. L. , Schwendinger J. , and Dillingham R. , Three-Year Follow-up of Positive Links: Higher Use of mHealth Platform Associated With Sustained HIV Suppression, AIDS and Behavior. (2024) 28, no. 8, 2708–2718, 10.1007/s10461-024-04405-z. 38869759 PMC11286697

[16] Reif L. K. , Abrams E. J. , Arpadi S. et al., Interventions to Improve Antiretroviral Therapy Adherence Among Adolescents and Youth in Low- and Middle-Income Countries: A Systematic Review 2015–2019, AIDS and Behavior. (2020) 24, no. 10, 2797–2810, 10.1007/s10461-020-02822-4. 32152815 PMC7223708

[17] Piran C. M. , Magalhães L. G. , Shibukawa B. M. , Rissi G. P. , and Merino M. D. , Treatment Non-Adherence or Abandonment Among Adolescents and Young Individuals Living With HIV/AIDS: A Scoping Review, Aquichan. (2023) 23, no. 2, 1–21, 10.5294/aqui.2023.23.2.2.

[18] Mokhele I. , Evans D. , Schnippel K. , and Long L. , Retention in HIV Care and Adherence to Antiretroviral Therapy in South Africa: A Systematic Review of Interventions Addressing Barriers Such as Stigma, Transport, and Psychosocial Support, Journal of the International AIDS Society. (2019) 22, no. S1.

[19] Muhula S. O. , Gachohi J. M. , Kombe Y. , and Karanja S. M. , Interventions to Improve Early Retention of PLWHs in Antiretroviral Therapy Programmes in Sub-Saharan Africa: A Systematic Review, PLoS One. (2022) 17, no. 2, 10.1371/journal.pone.0263663. PMC8827476 35139118

[20] Cloete C. M. , Hampton J. , Chetty T. et al., Evaluation of a Health System Intervention to Improve Virological Management in an Antiretroviral Programme at a Municipal Clinic in Central Durban, Southern African Journal of HIV Medicine. (2019) 20, no. 1, 10.4102/sajhivmed.v20i1.985. PMC6779997 31616575

[21] Umeokonkwo C. D. , Onoka C. A. , Agu P. A. , Ossai E. N. , Balogun M. S. , and Ogbonnaya L. U. , Retention in Care and Adherence to HIV and AIDS Treatment in Anambra State Nigeria, BMC Infectious Diseases. (2019) 19, no. 1, 10.1186/s12879-019-4293-8, 2-s2.0-85069766079. PMC6647106 31331280

[22] Maulsby C. H. , Ratnayake A. , Hesson D. , Mugavero M. J. , and Latkin C. A. , A Scoping Review of Employment and HIV, AIDS and Behavior. (2020) 24, no. 10, 2942–2955, 10.1007/s10461-020-02845-x. 32246357 PMC7716244

[23] Dessie G. , Wagnew F. , Mulugeta H. et al., The Effect of Disclosure on Adherence to Antiretroviral Therapy Among Adults Living With HIV in Ethiopia: A Systematic Review and Meta-Analysis, BMC Infectious Diseases. (2019) 19, no. 1, 10.1186/s12879-019-4148-3, 2-s2.0-85067499118. PMC6580562 31208346

[24] Habibi M. , Rahardjo S. S. , and Murti B. , Associations Between HIV Status Disclosure, Social Support, and Adherence to and Antiretroviral Therapy in Adults PLWHs With HIV/AIDS, Journal of Epidemiology and Public Health. (2021) 6, no. 1, 112–124, 10.26911/jepublichealth.2021.06.01.11.

[25] Monroe A. K. , Happ L. P. , Rayeed N. et al., Clinic-Level Factors Associated With Time to Antiretroviral Initiation and Viral Suppression in a Large Urban Cohort, Clinical Infectious Diseases: An Official Publication of the Infectious Diseases Society of America. (2019) 71, no. 7, e151–e158, 10.1093/cid/ciz1098. PMC7583410 31701144

